# Assessment of a novel antifungal ionic liquid-mediated silicone denture base soft liner

**DOI:** 10.3389/fdmed.2026.1734528

**Published:** 2026-02-18

**Authors:** Nadia Munir, Raja Azman Raja Awang, Naveed Inayat, Ghazala Suleman, Nawshad Muhammad, Noor Huda Ismail, Muhammad Sohail Zafar, Muhammad Zeeshan Munir, Abdul Samad Khan

**Affiliations:** 1 Department of Dental Materials, Lahore Medical and Dental College, University of Biological and Applied Sciences (UBAS), Lahore, Pakistan; 2 School of Dental Sciences, Universiti Sains Malaysia, Kubang Kerian, Kelantan, Malaysia; 3 Department of Periodontics, School of Dental Sciences, Health Campus, Universiti Sains Malaysia, Kubang Kerian, Kelantan, Malaysia; 4 Department of Prosthodontics, Azra Naheed Dental College, Superior University, Lahore, Pakistan; 5 Department of Prosthodontics, King Khalid University College of Dentistry, Abha, Saudi Arabia; 6 Department of Dental Materials, Institute of Basic Medical Sciences, Khyber Medical University, Peshawar, Pakistan; 7 Department of Prosthodontics, School of Dental Sciences, Health Campus, Universiti Sains Malaysia, Kubang Kerian, Kelantan, Malaysia; 8 Department of Clinical Sciences, College of Dentistry, Ajman University, Ajman, United Arab Emirates; 9 Center of Medical and Bio-allied Health Sciences Research, Ajman University, Ajman, United Arab Emirates; 10 School of Dentistry, University of Jordan, Amman, Jordan; 11 Discipline of Clinical Pharmacy, School of Pharmaceutical Sciences, Universiti Sains Malaysia, Penang, Malaysia; 12 Institute of Pharmaceutical Sciences, University of Veterinary and Animal Sciences, Lahore, Pakistan; 13 Department of Restorative Dental Sciences, College of Dentistry, Imam Abdulrahman Bin Faisal University, Dammam, Saudi Arabia

**Keywords:** antifungal activity, biomaterials, CD, ionic liquid, oral candidiasis, silicone soft denture liners

## Abstract

**Introduction:**

This *in vitro* study aimed to develop a novel choline-based ionic liquid-incorporated silicone denture base soft liners (SDBSLs) with improved antifungal potential and compliance in standard properties.

**Methods:**

Choline borate (CB) ionic liquid was synthesized, characterized, and blended with a silicone liner (Molloplast-B) in concentrations of 1%, 2%, and 5% w/w to form the experimental group, CB, with three subgroups (CB1%, CB2%, and CB5%, respectively). Commercial silicone liners served as the negative control, while silicone liners blended with the antifungal drug itraconazole (1.25% wt/wt) were the positive control. The antifungal potential against *Candida albicans* was evaluated via a direct-contact assay, with growth inhibition quantified by measuring the culture turbidity (OD600). The tear strength was determined using the type A test and the Shore A hardness was measured using a durometer. A physical parameter, percent mass change (%), was tested using the immersion method (at 1 week and 6 weeks). The Alamar Blue assay was performed to assess the biocompatibility of the samples.

**Results:**

Data were statistically analyzed using SPSS 25, with *p* = <0.001. The results showed that CB1% had the most significant inhibitory effect on *C. albicans* growth (83.71%). The drug- incorporated liners gained the most weight (5.00 ± 0.11%). The liners in CB5% were the toughest, with a tear strength of 5.31 ± 1.84 kN/m. The liners in the negative control group (Molloplast-B) were the softest (Shore A = 60.16 ± 1.07), followed by those in CB5% (51.33 ± 1.40). The biocompatibility of the ionic liquid-modified samples was better than that of the positive controls.

**Conclusion:**

The novel choline borate in silicone liners enhanced the antifungal potential and met the standard criteria of physical and mechanical compliance.

## 1 Introduction

The rising global incidence of oral diseases in denture wearers, especially denture stomatitis (DS), poses a considerable clinical and public health issue. Epidemiological studies show that 60%–65% of patients with complete dentures experience this *Candida albicans*-associated inflammatory condition, particularly those among the elderly and immunocompromised populations (1, 2). The pathogenesis entails intricate interactions between microbial biofilm formation on denture surfaces, host immune responses, and material-related factors (3). Silicone-based soft liners are clinically beneficial as they distribute masticatory forces and enhance comfort in patients with atrophic ridges; however, their inherent porosity and surface roughness paradoxically exacerbate this issue (4). These material characteristics facilitate optimal conditions for *C. albicans* adhesion and biofilm maturation, while concurrently undermining the mechanical integrity of the liner over time (5).

Current strategies for alleviating oral candidiasis have substantial challenges. Systemic and topical antifungals are effective in the short term, but they have disadvantages, such as drug resistance, patient compliance, and recurrence rates that are higher than 60% when treatment stops (6, 7). Changes to the materials to incorporate traditional antimicrobial agents (such as nystatin or fluconazole) or metal nanoparticles frequently worsen these significant physical and mechanical properties. For example, they may reduce flexural strength (8), bond strength (6), and viscoelasticity, and they may also increase water absorption up to 40%. This has led to a dire need for innovative biomaterial approaches that can halt microbial colonization while maintaining or enhancing the functionality of the material (8, 9). This research sought to develop a potent antifungal silicone liner that was intended not to replace standard care but to serve as a specific therapeutic adjunct. This material offers sustained local antifungal activity that minimizes the fungal reservoir in dentures during the temporary relining phase and potentially has long-term applications in at-risk patients such as the elderly, those who are immunocompromised, or those suffering from xerostomia. It is anticipated that choline borate (CB) ionic liquid (IL)-mediated denture liners may interrupt the cycle of reinfection and recurrence.

Choline-based ILs are a novel approach to denture liner remodeling, with specific benefits over conventional modifications. The choline cation is very biocompatible (cytotoxicity <10% at therapeutic dosages), whereas the borate anion has broad antibacterial action via disrupting membranes and inhibiting biofilm-related gene expression (10). Furthermore, the boric acid anion demonstrates broad-spectrum antifungal action, particularly against *C. albicans* and *Candida glabrata*, via multiple mechanisms (11). It breaks fungal cell membranes, inhibits important enzymes such as elastase, and prevents cell wall synthesis. In addition, its antioxidant properties aid in reducing oxidative stress in infected tissues. It is particularly effective for treating cutaneous candidiasis due to its multifunctional effects (12). The antifungal efficacy of choline borate has been established in cutaneous models, and its multi-target mechanism is pertinent for oral candidiasis to a certain extent. The disruption of membranes and inhibition of enzymes are essential strategies for addressing the intricate *Candida* biofilms that develop on mucosal surfaces in the oral cavity.

To maximize both biological activity (MIC90 values of 2–8 μg/mL against *C. albicans*) and material compatibility, the physicochemical characteristics of choline-based ionic liquids can be precisely adjusted through modification of the anion-cation combinations (13). According to recent developments, these materials can be successfully incorporated into medical polymers to enhance antimicrobial performance without sacrificing mechanical strength (14, 15).

The primary interaction between choline borate and the polydimethylsiloxane (PDMS) network of the silicone liner is facilitated by ion-dipole and hydrogen-bonding interactions, allowing for a stable dispersion within the silicone matrix. This incorporation strategy enables the controlled surface migration and sustained release of the choline borate ionic liquid, targeting the *Candida* biofilm. While direct studies on choline borate in PDMS are scarce, the principles of the interaction are well established (16). While effective first-line treatments exist for acute infection, there is a critical unmet need for dental biomaterials that provide sustained, localized antifungal activity as an adjunct to conventional therapy.

Novel ionic liquid-modified silicone liners are designed to fulfill this role. They are not intended to replace potent topical agents for eradicating established biofilms but rather to act as a preventive barrier, suppressing fungal recolonization of the denture surface following clinical treatment and thereby directly reducing the high recurrence rates associated with this condition.

In the literature, the mechanical properties of antifungal denture liners frequently deteriorate due to modifications. New dental biomaterials that can combat infection without compromising compliance are thus required. Ionic liquids based on choline offer a promising solution as an adjunct therapy in high-risk patients. They have established antimicrobial and material-enhancing properties. Although they have a plasticizing effect on polydimethylsiloxane, the incorporation does not affect compliance in terms of the standard performance criteria. Thus, the aim of this study was to formulate and assess the integrated mechanical and antifungal efficacy of silicone denture liners impregnated with these ionic liquids.

## 2 Methodology

### 2.1 Synthesis of choline-borate ionic liquid

Choline borate ionic liquid was synthesized via acid-base neutralization between equimolar aqueous solutions of choline hydroxide 46% (Sigma Aldrich, UK) and boric acid (Merck, Germany), following an established protocol (16). The reaction proceeded at 23 ± 2°C with constant stirring (300 rpm) for 12 h. Rotary evaporation (0.5 bar, 232°C, 6 h) and vacuum drying (0.1 bar, 70°C, 24 h) were both employed to concentrate the resulting solution. The final outcome was a viscous, dark brown liquid with distinctive ionic characteristics. The ionic liquid was promptly moved to an amber glass bottle with a polytetrafluoroethylene (PTFE)-lined septum due to it being hygroscopic. Before being characterized, the synthesized material was preserved in a desiccator.

### 2.2 Characterization of the choline borate ionic liquid

Fourier transform infrared spectroscopy (FTIR) and nuclear magnetic resonance (NMR) were employed to structurally characterize the synthesized choline borate ionic liquid.

An attenuated total reflectance (ATR) accessory for FTIR spectroscopy (Nicolet 6700-Wotol, United States) was utilized in the 4,000–650 cm^−1^ spectral range.

Each sample underwent 256 scans at a resolution of 8 cm^−1^. Molecular-level structural data in D_2_O solution were acquired using H-NMR (Bruker Avance, Billerica, United States) at 400 MHz.

### 2.2 Preparation of the composites

As indicated in Table 1, the experimental design comprised five study groups: three experimental groups, a negative control, and a positive control. This configuration made it possible to compare novel formulations to baseline and therapeutic reference standards. Commercial silicone liner Molloplast-B (45 g) dough was divided into three 15 g parts. Prior to the specimen preparation, the material was homogenized on non- absorbent pads for 2 to 3 minutes and the precise amounts were measured using a Shimadzu electronic balance. To achieve homogeneous experimental mixes, each piece was manually kneaded at room temperature with choline borate IL at concentrations of 1%, 2%, or 5% to constitute the experimental groups (CB1%, CB2%, and CB5%). Positive controls were prepared by kneading 15 g of Molloplast-B dough with 1.25% w/w itraconazole. The negative controls had no additive. The doughs were then stored in five labeled containers according to study group. After the preparations, the samples were processed by 2 h of heat curing in molds at 74 °C in a water bath and subjected to 1 h of terminal boiling. After the cured samples had cooled to room temperature in the water, they were removed from the molds.

**Table 1:** Distribution of the study groups.

Groups	Composition
Negative control [Molloplast-B; commercial silicone denture liners (SDBSLs)]	Molloplast-B (commercially available SDBSLS)
Positive control (drug incorporated SDBSLs)	1.25% itraconazole-impregnated Molloplast-B
CB1% (1% choline borate-incorporated SDBSLs)	Molloplast-B + 1% CB ionic liquid
CB2% (2% choline borate-incorporated SDBSLs)	Moloplast-B + 2% CB ionic liquid
CB5% (5% choline borate-incorporated SDBSLs)	Moloplast-B + 5% CB ionic liquid

### 2.3 Antifungal activity assessment

The antifungal efficacy of the modified silicone liners against *C. albicans* (ATCC 10231) was assessed using a broth microdilution turbidimetric assay to quantify the percentage inhibition of planktonic fungal growth. This method measures the suppression of growth in a suspension upon direct and continuous contact with the test material. Disc-shaped specimens (6 mm diameter × 2 mm thickness) from each study group were sterilized by immersion in 70% ethanol for 5 min and air-dried under aseptic conditions. Each sterilized specimen was aseptically transferred to a sterile 1.5 mL microcentrifuge tube containing 0.5 mL of sterile brain heart infusion (BHI) broth.

A standardized fungal inoculum was prepared from a fresh culture by adjusting its turbidity in sterile saline to match a 0.5 McFarland standard (approximately 1.5 × 10⁸ CFU/mL).

Subsequently, 200 µL of this standardized inoculum was added to each tube containing a specimen and broth, ensuring direct contact. The tubes were then incubated at 37°C for 1 h under static conditions to allow initial microbial interaction with the material surface.

Following this incubation period, 0.5 mL of fresh, sterile BHI broth was added to each tube, bringing the total volume to 1.0 mL. The initial optical density at 600 nm (OD₆₀₀) of each tube was recorded (*T* = 0 h) using a UV-Vis spectrophotometer. The tubes were then incubated aerobically for 24 h at 37 °C. After incubation, the final OD₆₀₀ was measured (*T* = 24 h).

The negative control consisted of tubes containing the standardized inoculum and broth without any test specimen. A positive growth control was included to verify inoculum viability, and a sterility control (broth only) confirmed the aseptic technique. The percentage inhibition of fungal growth for each test specimen was calculated using the following formula:

$$\mathrm{Percent}\;\mathrm{inhibition}=\frac{\left(\mathrm{OD}\;\mathrm{of}\;\mathrm{control}\;\mathrm{after}\;\mathrm{incubation})-(\mathrm{OD}\;\mathrm{of}\;\mathrm{sample}\;\mathrm{after}\;\mathrm{incubation}\right)}{\mathrm{OD}\;\mathrm{control}\;\mathrm{after}\;\mathrm{incubation}}\;\times 100$$

All the experimental procedures were performed in triplicate, and mean values with standard deviations are reported. Sterilization of tubes, broth, and instruments was achieved by autoclaving at 121°C for 15 min prior to use.

### 2.4 Mechanical testing

The sample size was calculated as follows (Table 2).

**Table 2:** Sample size estimation for the study groups.

Test method	Standard/reference applied	Sample number *(n)*
Mechanical testing (tear strength, tensile Bond strength, and hardness)	Tear strength (ISO 34-1, 2015)	(*n* = 6)
	Tensile bond strength (ISO 10139-2., 2016)	(*n* = 6)
	Hardness (ISO 7619)	(*n* = 6)
Physical testing (percent mass change)	Water sorption (ISO 10139-2)	(*n* = 5)

#### 2.4.1 Shore A hardness assessment

For the Shore A hardness test, five disc-shaped specimens 6 mm in diameter and 2 mm in height were prepared. A WESTOP Type A durometer (Nishi Tokyo Seimitsu Co. Ltd., Japan) was used to measure hardness in compliance with the International Standards Organization (ISO) 7619-1 guidelines (17). Prior to testing, all the samples were conditioned for 24 h by immersion in distilled water at 37 °C. Each specimen was placed on a flat, rigid surface after incubation, and the durometer's indenter was gradually lowered until contact was made. To ensure consistent results, measurements were made following a 5 s loading period. Each sample was evaluated at three different points, and the mean values were recorded for further analysis (18).

#### 2.4.2 Tensile bond strength evaluation

**Figure 1:**
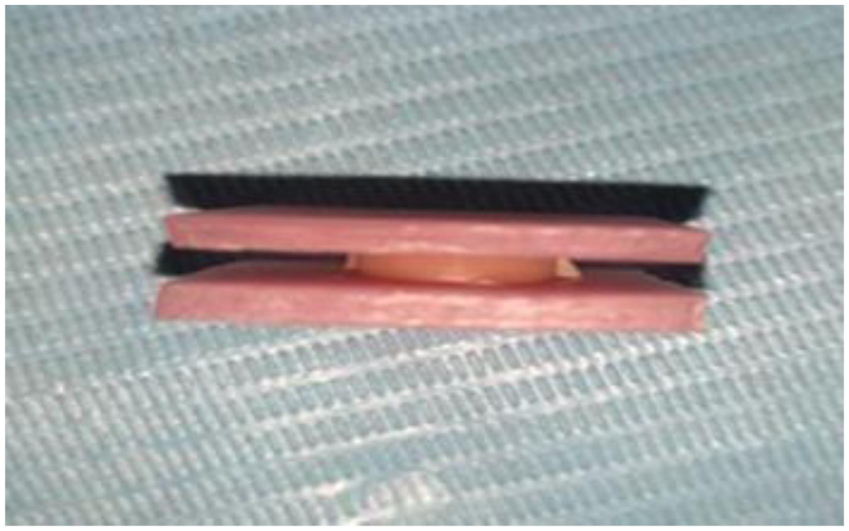
Tensile bond strength test assembly. Heat-cured polymethylmethacrylate (PMMA) plates are bonded to silicone denture soft liners (SDBSL).

Each sample had its tensile bond strength evaluated in accordance with the ISO 10139-2 guidelines (19, 20). In order to replicate the denture base-acrylic interface, the test assembly was made up of two heat-cured acrylic plates (7 mm × 7 mm × 2 mm) that were bonded to the 6 mm × 2 mm Molloplast silicone denture base soft liner (SDBSL) discs (Figure 4). To remove uneven surfaces, the plates were abraded using a 500-grit abrasive material. The silicone liner adhesive (Primo adhesive; DETAX GmbH & Co. K, Germany) was applied to the working surface of the prepared heat-cured acrylic plates prior to the bonding. For mechanical stability during testing, a self-cured acrylic handle measuring 76.2 mm × 25.4 mm × 3 mm was fastened to each acrylic plate. To measure the tensile bond strength, the assembly was clamped vertically in the jig of a universal testing machine (UTM; AG-X Plus, Shimadzu, Japan) (Figure 1).

The UTM recorded the maximum force (N) prior to bond failure. A vernier caliper was used to measure each specimen's cross-sectional area to calculate the tensile bond strength (MPa). The maximum recorded force (N) divided by the bonded area (mm^2^) yielded the tensile bond strength in MPa.

After being clamped between the testing machine's jaws, the sample was stretched at a rate of 10 mm/min and at load cell capacity until it ruptured completely. Subsequently, the tensile bond strength, *σ*B (MPa), was determined using the following formula:

$$\begin{array}{cc} \sigma B=\; & \mathrm{Fmax}\\ & A \end{array}$$

where Fmax = maximum recorded force expressed in N; A = an initial area of the silicone connection or the cross-section of silicone layers in a plane parallel to the base of the sample with acrylic.

#### 2.4.3 Tear strength testing

**Figure 2:**
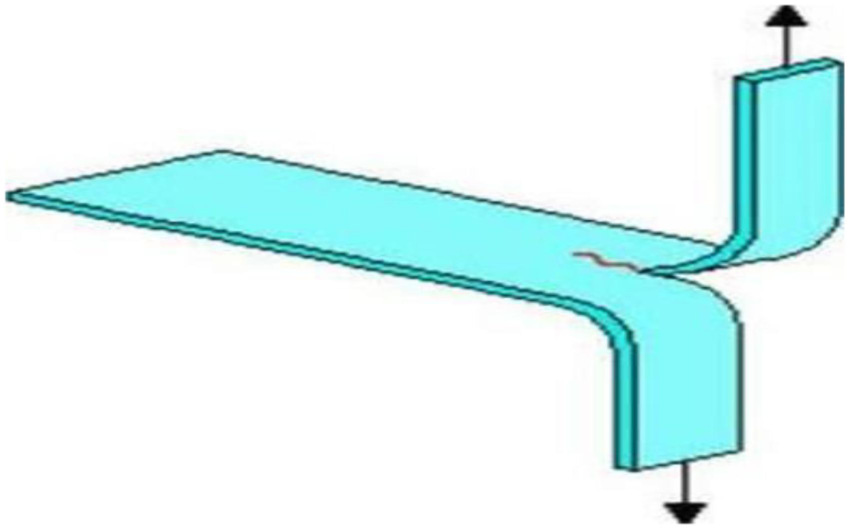
Simulation of a trouser tear specimen in the pretest setup phase.

Test specimens in the form of trousers were created for each group using a PTFE split mold measuring 100 mm × 15 mm × 2 mm. They were heat-cured for 2 h at 74° and then terminally boiled for an hour in a water bath. A surgical blade was then used to cut a 60 mm central incision in each specimen, creating two symmetrical legs (Figure 2). The legs were tested by mounting them vertically in opposing axial alignment in the grips of a universal testing machine (Shimadzu AG-X Plus, Tokyo, Japan). According to ISO 34-1:2015, Type C Test, the tear strength (Ts) was computed as the median tearing force (F) divided by the specimen thickness (d), and the result was expressed in kN/m (21).

#### 2.4.4 Physical property assessment (percent weight change)

**Figure 3:**
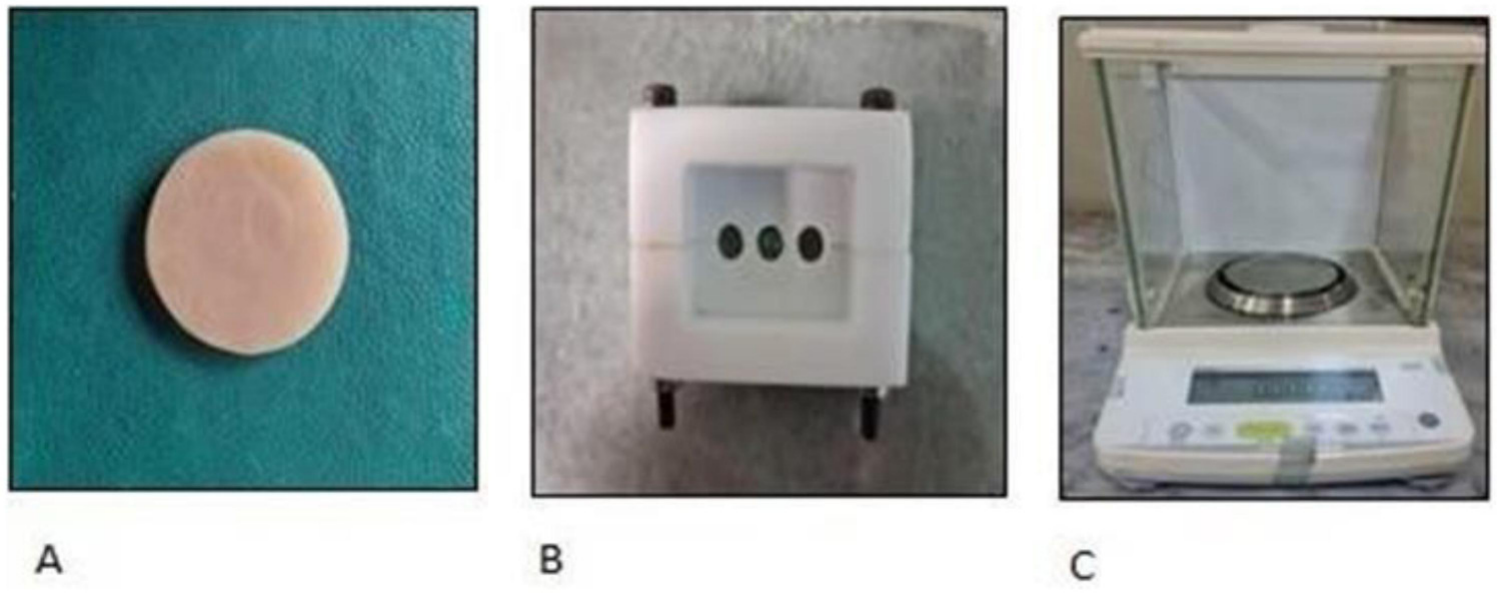
Sample assembly for percent weight assessment. **(A)** Disc samples of silicone denture liners; **(B)** PTFE mold; and **(C)** analytical balance.

Disc samples from each group were preconditioned by drying in a desiccator with silica gel at 37°C and 23°C to establish a constant baseline mass (M1), which was weighed using a Shimadzu electronic balance (TXL 323L Tokyo, Japan) with an accuracy of 0.0005 g (Figure 3).

After initial immersion in distilled water at 37°C and pat drying, their mass was recorded at 1-week (M2) and 6-week (M3) intervals on the same analytical balance to calculate water sorption. Following immersion, the samples were re-dried at each interval in a desiccator until a constant mass was reached. These mass values (M and M1) were used to calculate the final percentages of water net mass change according to the following established formulae:

$$\mathrm{Weight}\;\;\mathrm{change}\;\;(\text{\%})\mathrm{after}\;1\;\mathrm{week}\;=\;\frac{\left(M2\text{-}M1\right)}{M1}\;\times 100$$

where M is the mass after one week of immersion and M_1_ is the initial mass before immersion; and

$$\mathrm{Weight}\;\;\mathrm{change}\;\;(\text{\%})\mathrm{after}\;6\;\mathrm{weeks}\;=\;\frac{\left(M3\text{-}M1\right)}{M1}\;\times 100$$

where M_1_ is the initial mass before immersion, M2 is the mass after 6 weeks of immersion, and M3 is the mass after 6 weeks of immersion.

#### 2.4.5 Cell viability testing

The viability of NIH3T3 mouse fibroblast cells (ATCC CRL-1658, passage 20) cultured on the test materials for 7 consecutive days was assessed using an Alamar Blue assay. At 37 °C with 5% CO₂, cells were cultured in Dulbecco's Modified Eagle Medium (DMEM) supplemented with 10% fetal bovine serum (FBS) and 1% penicillin streptomycin (PenStrep). A 24-well plate was seeded with 8,000 cells per well for the assay, and the cells were left to adhere for 16 h. Sterilized samples were then added to the wells. The cells were cultured for 1, 4, and 7 days before being rinsed with phosphate-buffered saline (PBS) and incubated for 4 h with Alamar Blue working solution, which was created by diluting a 1 mM stock in culture media.

Following incubation, the solution was transferred to a 96-well plate, and the absorbance was measured at 570 nm using a plate reader (SpectraMax M2/M2e Microplate Readers, Molecular Devices). Cell viability was calculated relative to a sample-free control group, with the background absorbance subtracted using a blank only containing Alamar Blue solution. All the experiments were performed in triplicate.

### 2.5 Statistical analysis

The statistical analyses were conducted using SPSS software (version 25, IBM Software, Chicago, IL, USA). Normality was assessed using the Shapiro–Wilk test (*p* = 0.200), and a logarithmic transformation was applied to meet the assumptions of parametric tests. Transformed data are presented as mean ± standard deviation. Intergroup comparisons were performed using a one-way analysis of variance (ANOVA), followed by Tukey's *post hoc* test for multiple comparisons. Statistical significance was defined as *p* ≤ 0.05.

## 3 Results

### 3.1 Characterization of choline borate ionic liquids

NMR and FTIR analyses verified the ionic liquid's structure. The key evidence included FTIR bands corresponding to B-O stretching vibrations and the hydroxyl group of the choline moiety. H-NMR revealed a characteristic chemical shift consistent with the tetrahedral coordination of the boron atom.

### 3.2 Fourier transform infrared spectroscopy

**Figure 4:**
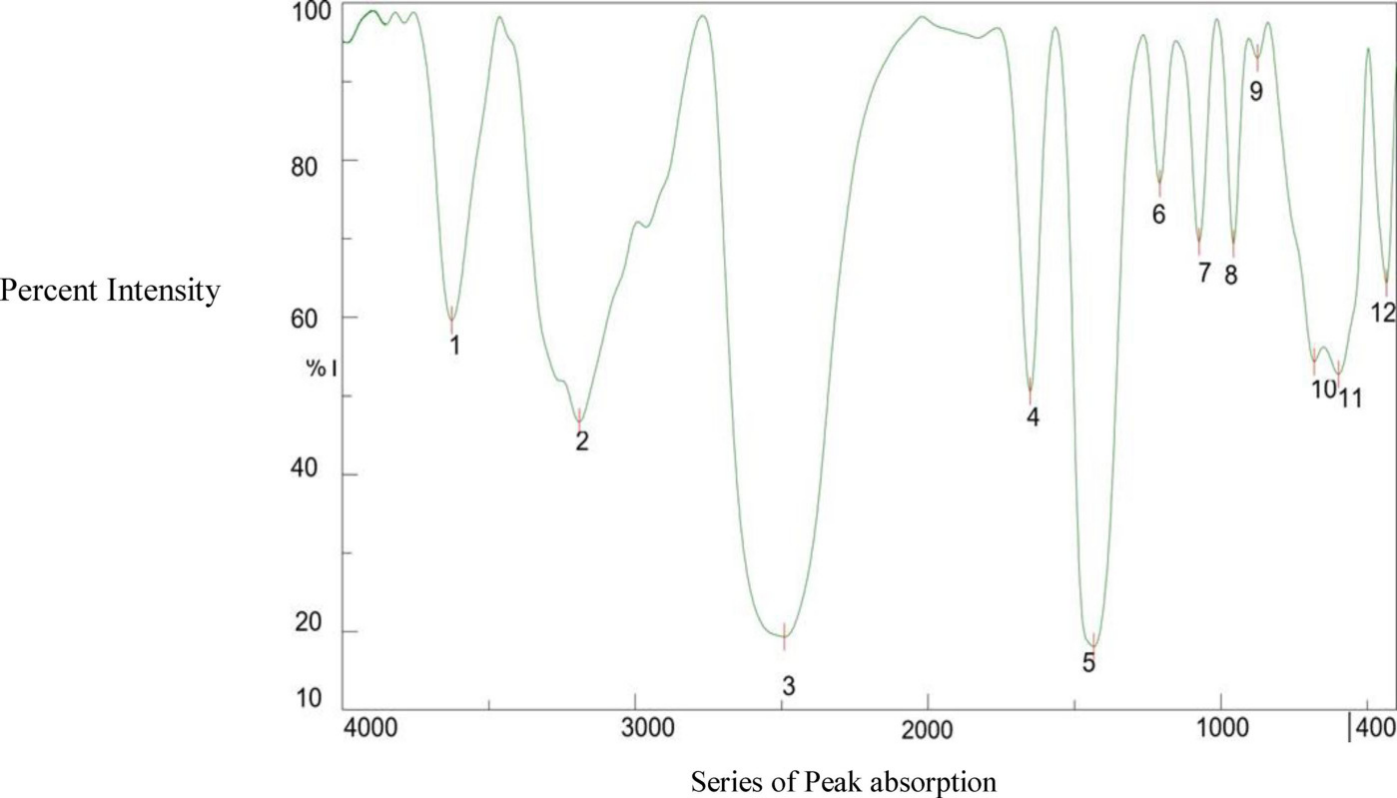
FTIR spectra of the choline borate ionic liquid.

O-H stretches from the borate and choline hydroxyls, which are frequently involved in hydrogen bonding, are indicated by a broad band in the 3,200–3,600 cm^−^^1^ region. Crucially, the C-N stretches of the choline cation are clearly visible between 1,350 and 1,450 cm^−^^1^ (B-O asymmetric) and approximately 1,000 cm^−^^1^ (B-O–C). The B-O stretches appear as strong, distinctive bands between 1,000 and 1,100 cm^−^^1^. The spectrum thus reflects a hybrid material featuring the ionic choline headgroup and the covalently bonded borate anion, as shown in the summary (Table 3) of peak assignments (22). The peak assignments are visible in Figure 4 and Table 3.

**Table 3:** Summary of peak assignments in the FTIR spectra from the choline borate ionic liquid.

Peak label	Approx. wavenumber (cm^−^^1^)	Assignment
1	∼3,350	Broad O-H stretch (B-OH, Choline- OH)
2	∼3,030	(Weak) Aromatic C-H stretch
3	∼2,960	C-H stretch, asym (-CH₃)
4	∼2,870	C-H stretch, sym (-CH₃)
5	∼1,485	C-H bending (CH₂, CH₃)
6	∼1,390	B-O stretch, asym (key band)
7	∼1,085	C-O stretch and B-O-C stretch
8	∼950	C-N stretch
9	∼880	B-O-H bending
10	∼720	CH₂ rocking
11	∼660	O-B-O bending
12	∼520	Skeletal deformation

### 3.3 Nuclear magnetic resonance

**Figure 5:**
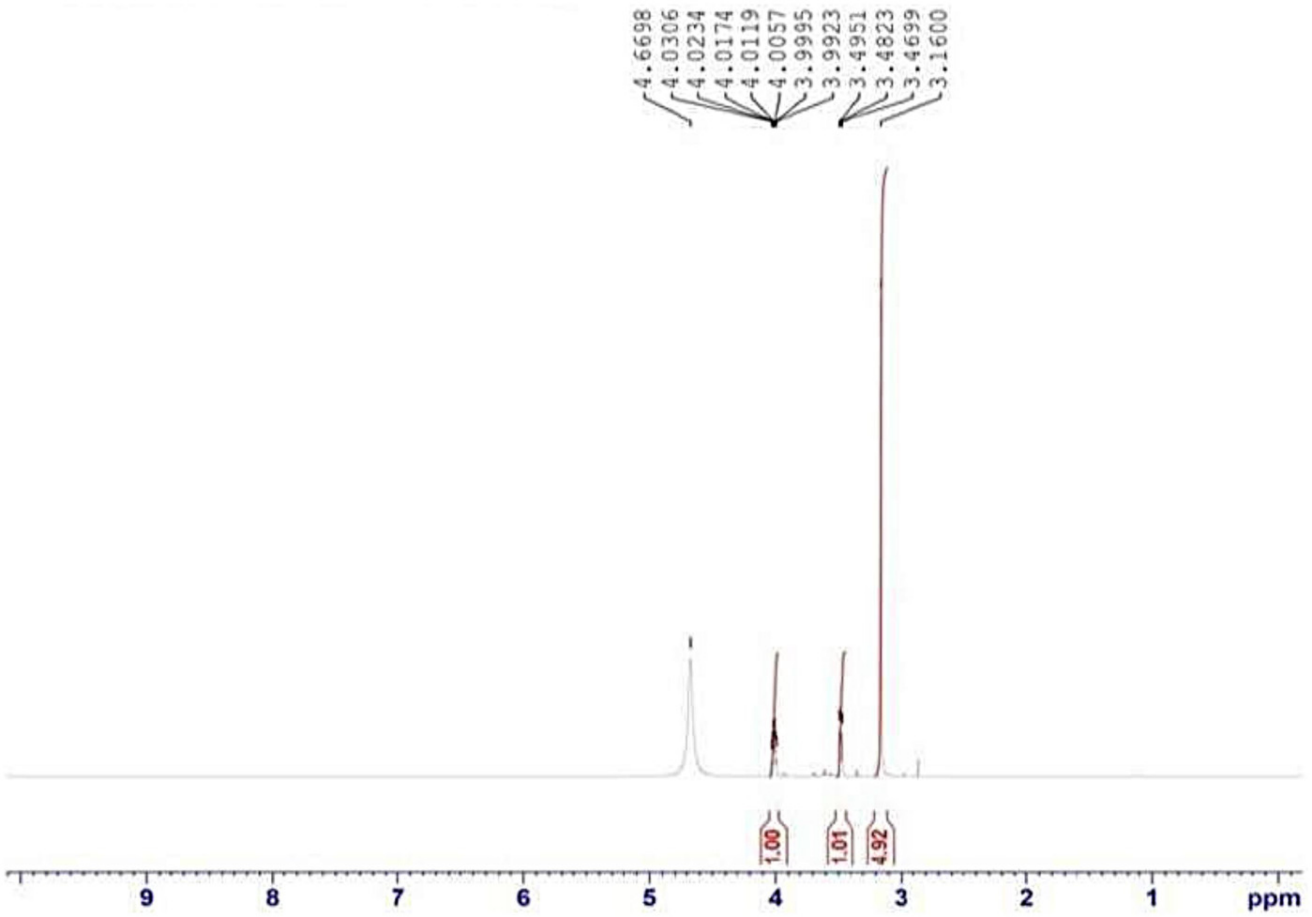
NMR spectra of the choline borate ionic liquid.

^1^H-NMR analysis confirmed the presence of the choline cation. The spectrum showed the following characteristic choline resonances: a sharp singlet at ∼3.16 ppm [9H, -N (CH₃)₃], a multiplet between ∼3.47–3.50 ppm (2H, -N-CH₂-), and a complex signal between ∼3.99-4.67 ppm (3H, -O-CH₂-CH₂-OH), which integrates to the expected proton ratio (Figure 5). The sample was dissolved in D₂O, as indicated. The borate anion (BO3^−^) does not produce distinct signals in the 1H-NMR spectrum due to its symmetry and lack of hydrogen atoms.

### 3.4 Antifungal testing

A direct contact assay was conducted to quantitatively assess the CB ionic liquid's antifungal activity against *C. albicans*. Growth suppression was effectively determined by calculating the percent inhibition, which was based on the decrease in optical density (OD₆₀₀) after 24 h of incubation in comparison to the initial OD (Table 4). The negative control group (Control -) exhibited limited antifungal activity, which was regarded as the baseline. The positive control group (Control +) exhibited moderate resistance to the growth of *C. albicans*.

**Table 4:** Comparison of mean OD values before and after the incubation of the samples using a paired sample *t*-test.

Group	Mean OD before incubation	Mean OD after incubation	Mean difference	*t*-Value
Control +	0.012 ± 0.0001	0.031 ± .0033	−0.0188	−10.016
Control −	0.148 ± 0.0008	0.156 ± .001	−0.008	−13.653
1% choline borate	1.784 ± 0.0017	0.019 ± .0001	1.7644	1,856.406
2% choline borate	0.302 ± 0.0056	0.039 ± .0126	0.2635	36.871
5% choline borate	0.028 ± 0.0005	0.043 ± .0054	−0.0143	−5.055

**Figure 6:**
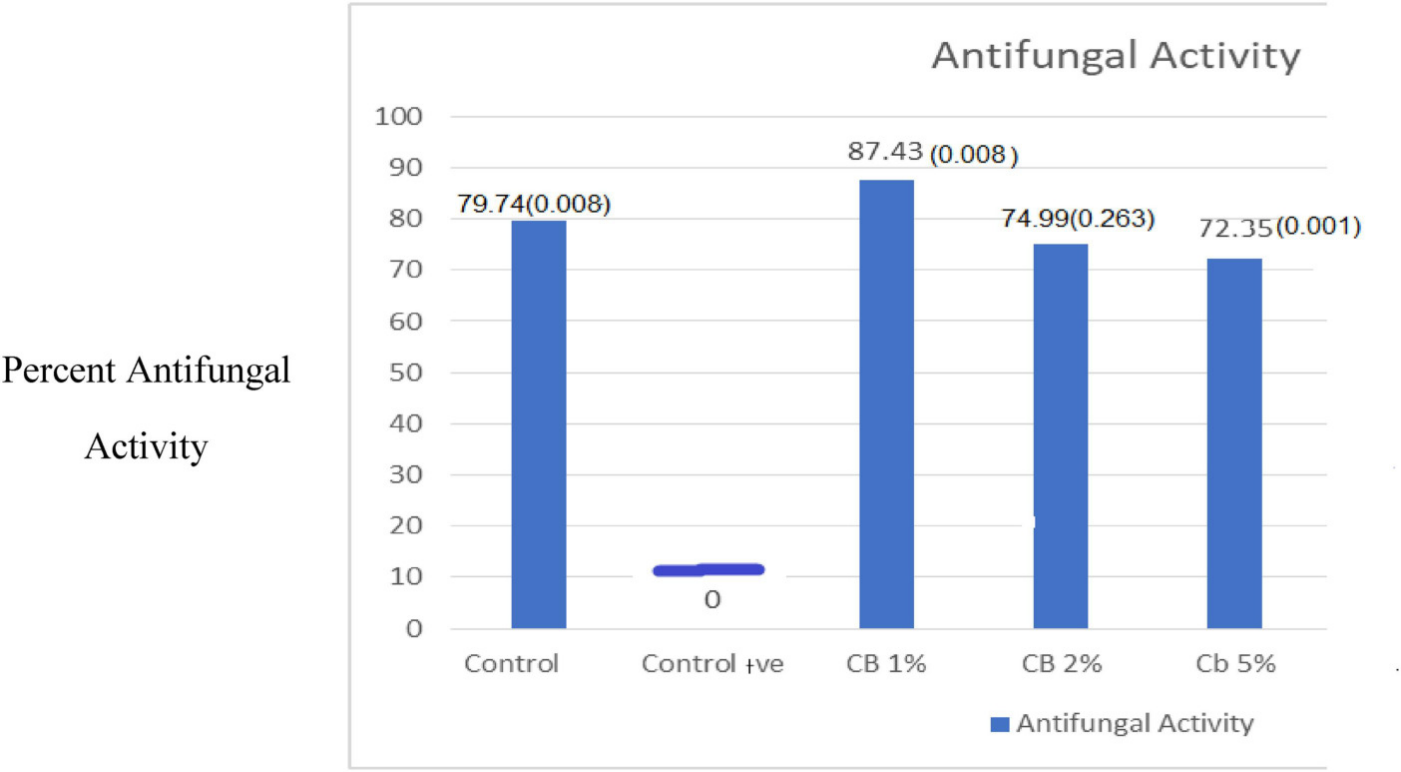
Comparison of antifungal activity percentages in the study groups.

The silicone liners with 1% CB exhibited the highest inhibition, with a large mean OD reduction of 1.76 and the highest percent inhibition of 87.43% (*p* < 0.001). The CB2 group also showed strong and significant activity, with a mean OD reduction of 0.2635 (*p* = 0.001), exhibiting 74.99% inhibition. In contrast, the higher CB 5% concentration demonstrated a negligible mean OD reduction (**−**0.0143), with either a potential decline of 72.35% in efficacy or the presence of a confounding factor at this concentration (Figure 6).

### 3.5 Shore A hardness assessment

**Figure 7:**
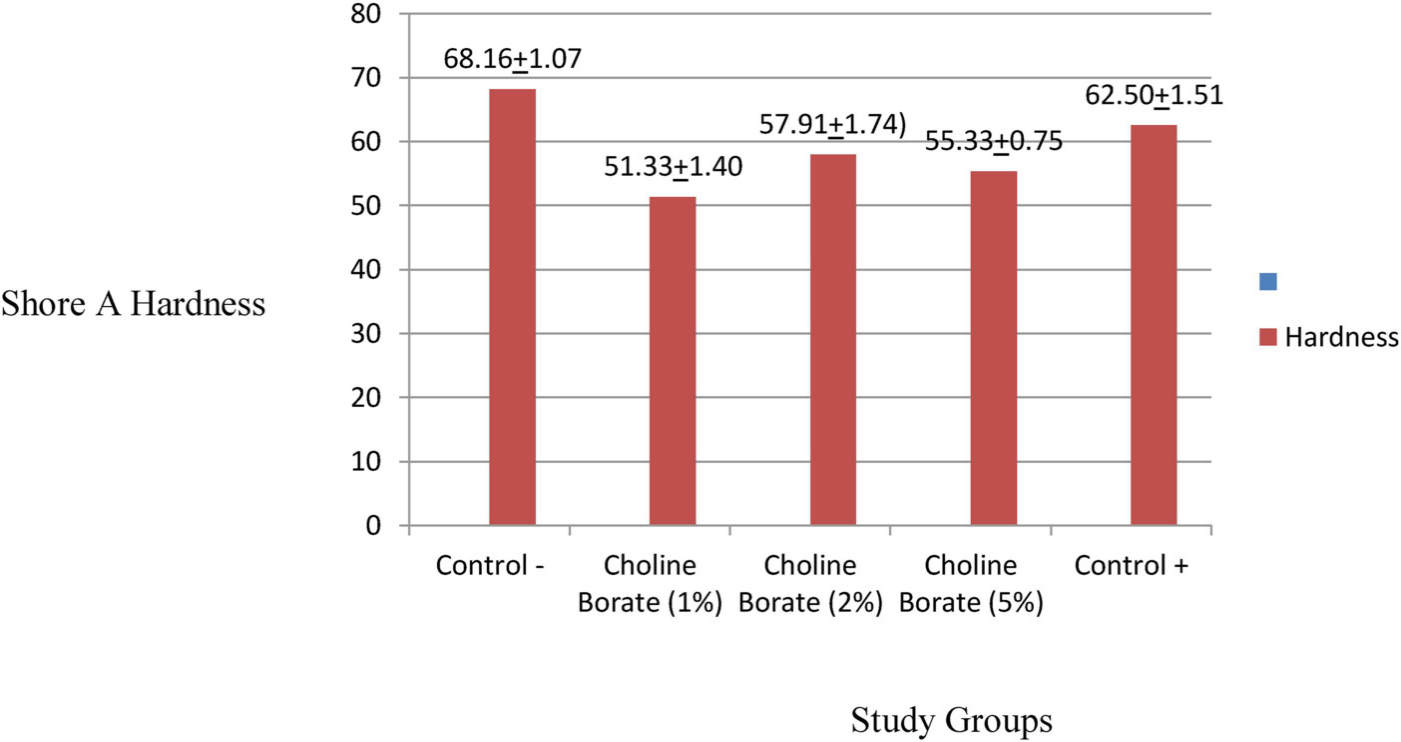
Comparison of shore A hardness in the study groups.

With a mean hardness value of 68.16 ± 1.07, the negative control group outperformed the positive control group by a significant margin (62.50 ± 1.51). The CB2% and CB5% concentrations exhibited comparable means (57.91 ± 1.74 and 55.33 ± 0.75, respectively), while the 1% concentration group was the most resilient (51.33 ± 1.40). All the experimental groups demonstrated lower hardness values and a soft texture (Figure 7).

A one-way ANOVA revealed that the treatment had a statistically significant effect on the samples' hardness (*p* < 0.001). The negative control group demonstrated significantly greater hardness compared to both the positive control and all experimental groups. Furthermore, the positive control group exhibited significantly greater hardness than all the experimental groups containing choline borate.

### 3.6 Tear strength

**Figure 8:**
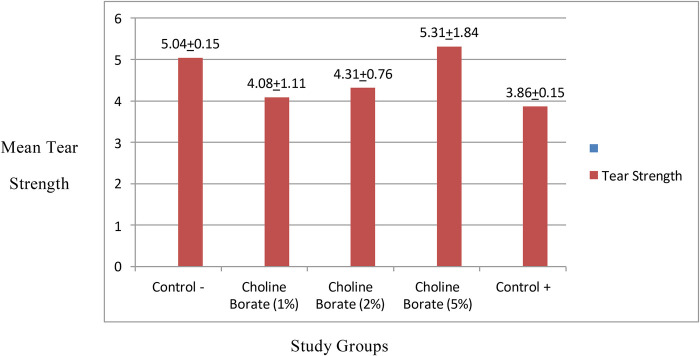
Comparison of tear strength in the study groups.

The treatment had a statistically significant effect on the tear strength of the samples (one-way ANOVA, *p* < 0.05). The negative control group demonstrated the greatest tear strength, with a mean value of 5.04 ± 0.15 kN/m. Among the experimental groups, the 5% choline borate concentration yielded the highest mean value (5.31 ± 1.84 kN/m), though with considerable variability. The 1% and 2% choline borate groups yielded intermediate mean tear strengths (4.08 ± 1.1 kN/m and 4.31 ± 0.76 kN/m, respectively). The positive control group demonstrated the lowest mean tear strength (3.86 ± 0.15 kN/m), as illustrated in Figure 8.

### 3.7 Tensile bond strength

The tensile bond strength (TBS) of the SDBSLs bonded to the acrylic resin base differed significantly across the experimental groups, as determined by a one-way ANOVA (*p* < 0.0001).

**Figure 9:**
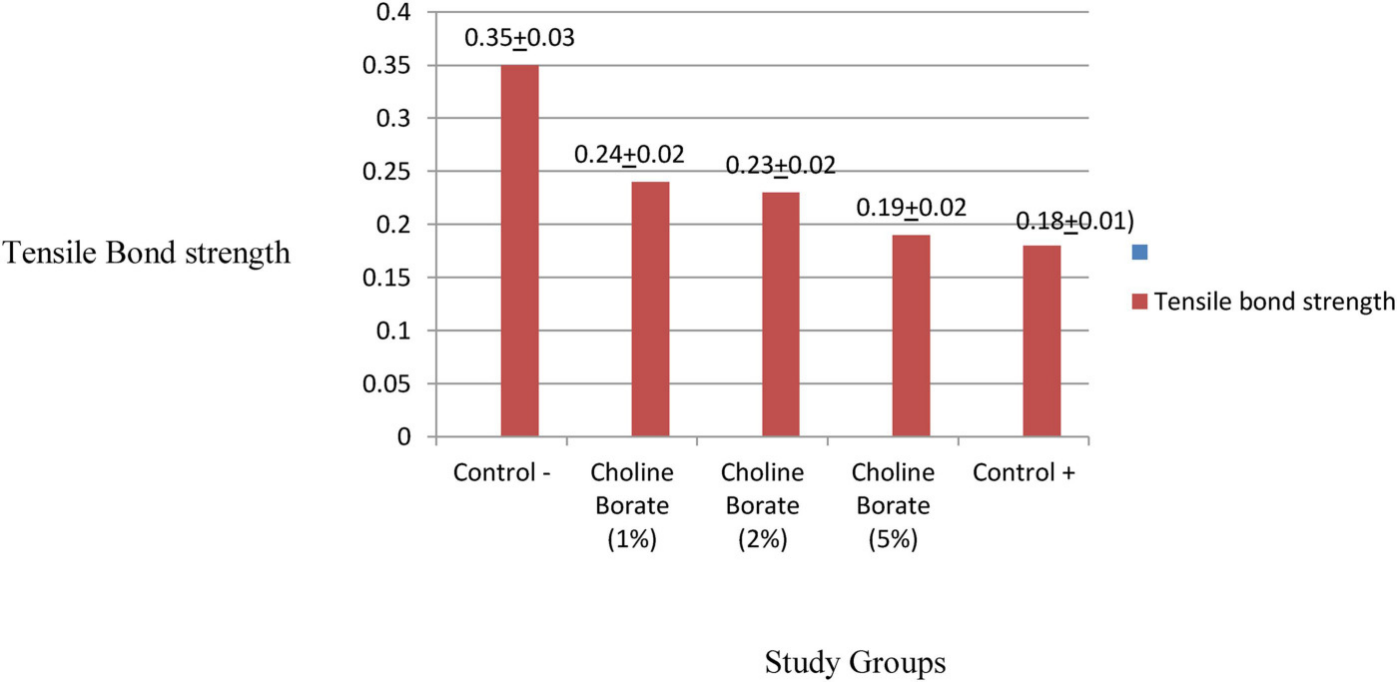
Comparison of tensile bond strength in the study groups.

The negative control group demonstrated the highest mean TBS (0.358 ± 0.036 MPa). The TBS of the experimental groups decreased with increasing concentrations of choline borate, with the 1%, 2%, and 5% groups showing means of 0.240 ± 0.027 MPa, 0.230 ± 0.027 MPa, and 0.190 ± 0.026 MPa, respectively. The positive control group exhibited the lowest mean TBS (0.180 ± 0.016 MPa). Figure 9 illustrates a comparison of tensile bond strength in the study groups. Critically, the mode of failure in all the groups was observed to be failure of the adhesive at the SDBSL-acrylic resin interface.

The mechanical property data (Table 5) clearly show that choline borate caused the liners to be more viscoelastic and reduced the material's hardness, with the 1% concentration lowering it from 68.2 to 51.3. The values were compliant with the standard criteria for the hardness of soft liners. Most notably, CB causes a steep, dose-dependent decrease in TBS, which fell from 0.358 to 0.190 with the 5% concentration, performing similarly to the weak-bonding positive control (0.180). Its effect on tear strength, however, is inconsistent and not concentration-dependent.

**Table 5:** Data summary of the mechanical properties in the study groups.

Variable	Shore A hardness	Tear strength	Tensile bond strength
Control −	68.16 (1.07)	5.04 (0.15)	0.358 ± 0.036
1% choline borate	51.33 (1.40)	4.08 (1.11)	0.240 ± 0.027
2% choline borate	57.91 (1.74)	4.31 (0.76)	0.230 ± 0.027
5% choline borate	55.33 (0.75)	5.31 (1.84)	0.190 ± 0.026
Control +	62.50 (1.51)	3.86 (0.15)	0.180 ± 0.016

### 3.8 Percent mass change

**Figure 10:**
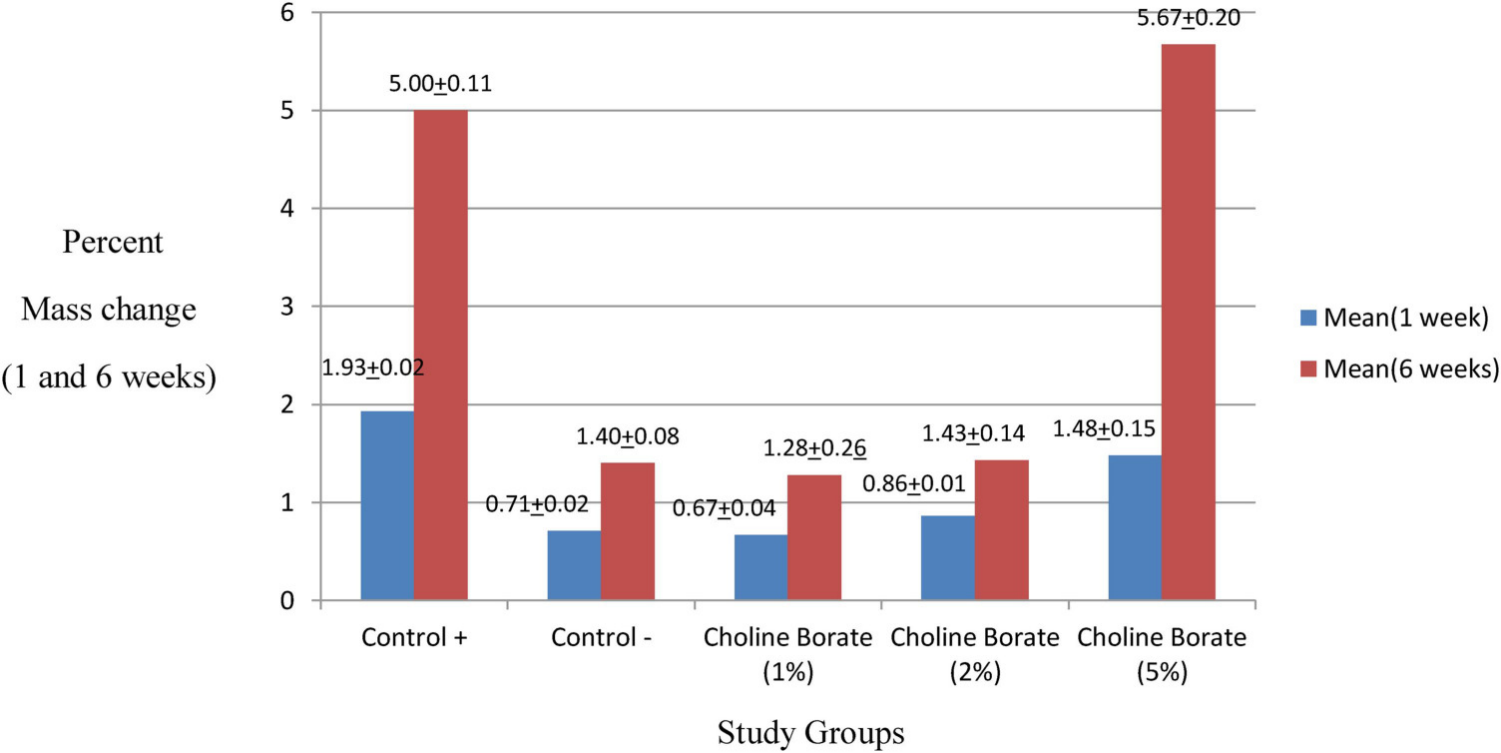
Comparison of percentage mass change after 1 week and 6 weeks of immersion.

Based on the comprehensive analysis of weight change over 6 weeks, the material stability was highly concentration-dependent, as shown in Table 6. The 5% CB formulation and Control+group exhibited poor stability, demonstrated by substantial mass increase at 1 week (1.48% ± 0.15 and 1.93% ± 0.02, respectively), followed by significant net mass loss by 6 weeks (−4.18% and −3.07%, respectively), indicating pronounced degradation and dissolution. In contrast, the 1% and 2% CB formulations demonstrated minimal mass change throughout the study period (0.67%–0.86% at 1 week; −0.57 to −0.61% net change at 6 weeks) that was statistically equivalent to the stable Control - group, confirming their superior structural integrity and resistance to aqueous interaction (Figure 10).

**Table 6:** Comparison of the percent mass change in the control and the study groups after 1 and 6 weeks of immersion.

Variable	*N*	Percent mass change		*P*
		Mean (1 week)	Mean (6 weeks)	
Control +	5	1.93(±0.02)	5.00(±0.11)	<0.001
Control −	5	0.71(±0.02)	1.40(±0.08)	
1% choline borate	5	0.67(±0.04)	1.28(±0.26)	
2% choline borate	5	0.86(±0.02)	1.43(±0.14)	
5% choline borate	5	1.48(±0.15)	5.67(+0.20)	

### 3.9 Cell viability

**Figure 11:**
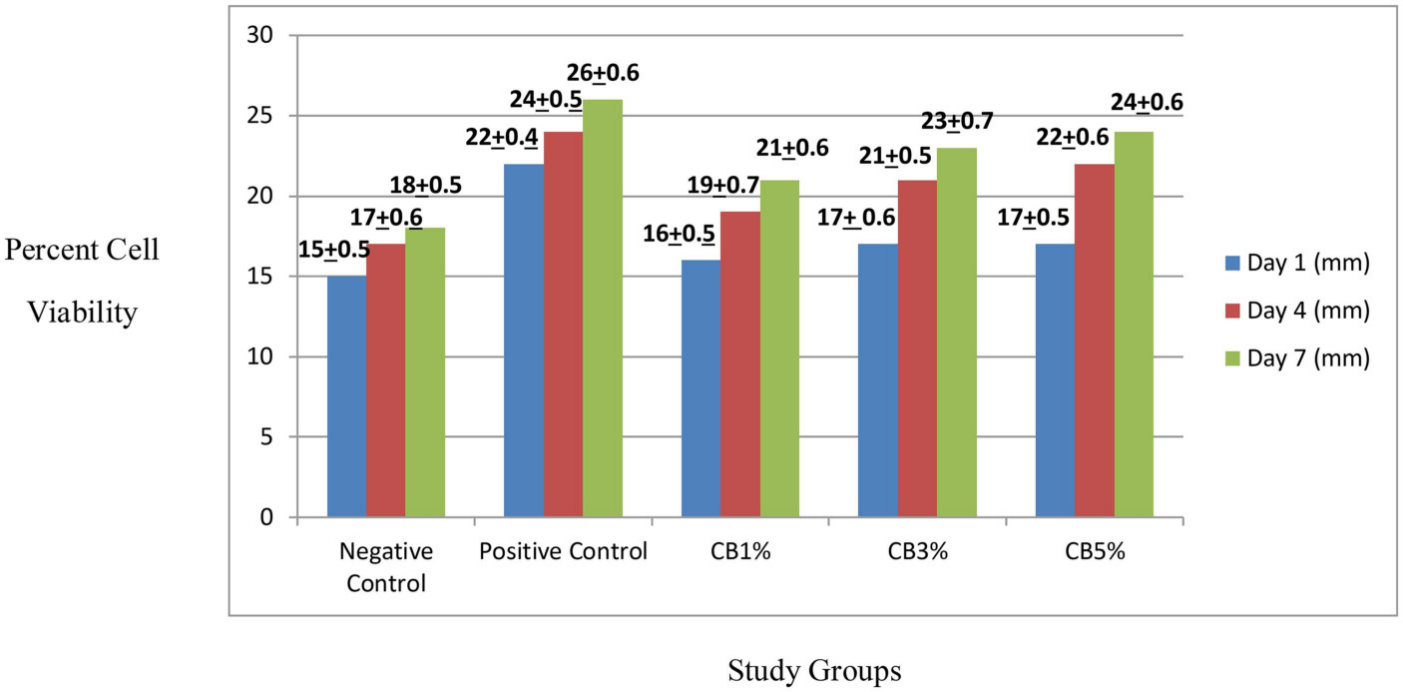
Percent cell viability in the study groups on days 1, 4, and 7.

**Figure 12:**
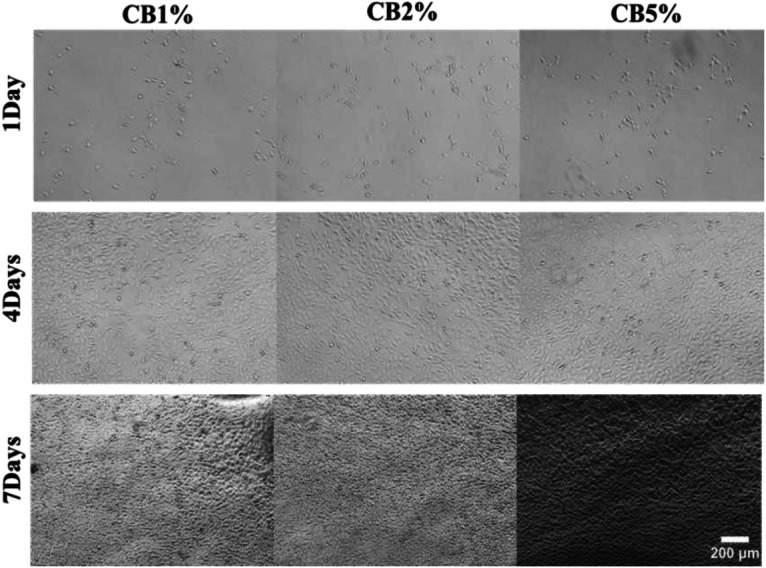
Representative phase-contrast images (10×) showing cell attachment and morphology on the different materials at days 1, 4, and 7.

The quantitative analysis of the Alamar Blue assay results demonstrated a steady rise in metabolic activity. Following a pattern similar to the positive control group and demonstrating a lack of early cytotoxicity, the CB1%, CB2%, and CB5% groups repeatedly showed greater proliferation on days 1 and 4 compared to the negative control. By day 7, proliferation values increased further across all samples; however, the increase was most notable in the CB1% and CB5% groups, which surpassed the control readings and corresponded with the previously identified statistically significant increases (*p* < 0.05). In contrast, although the CB2% group maintained higher activity than the negative control group throughout the study period, it did not demonstrate a distinct day-7 proliferation spike comparable to the CB1% and CB5% groups, as demonstrated in Figures 11, 12.

## 4 Discussion

The properties of silicone-based materials can be altered either positively or negatively by the incorporation of ionic liquids. According to previous studies, some types of ILs can enhance both the viscoelastic behavior and antifungal potential of denture liners, improving the patient's comfort. The effects of CB ionic liquid on the mechanical, biological, physical, and antifungal characteristics of silicone-based soft denture liners were thoroughly assessed in this study. The findings show that choline borate ionic liquid has a distinct, concentration-dependent impact on all the standard characteristics, indicating a crucial balance is required between material performance and therapeutic efficacy.

The strong, non-linear antifungal action against *C. albicans* is the most important finding. The choline borate ionic liquid at concentrations of 1% and 2% outperformed the controls with remarkable inhibition (87.43% and 74.99%, respectively). The antifungal properties of boric acid and the choline cation's combined membrane-disruptive action are responsible for this effectiveness (3).

This non-linear and non-concentration-dependent activity profile indicates that the choline borate ionic liquid exhibits optimized antifungal potential at lower concentrations (1% and 2%), significantly outperforming the control groups.

A negative difference in the optical density value from 24 h before and after incubation indicates a lower antifungal activity value, which is concentration independent. One possible reason for this is the leaching of choline borated ionic liquid at high concentrations (5%) and the possible loss of antifungal potential of the experimental liners in the CB5% group. The high concentration of hydrophilic choline borate may rapidly leach from the silicone matrix, increasing the ionic strength and turbidity of the broth (23). This may increase the *initial* optical density in the CB5% group, resulting in a smaller or negative OD difference after 24 h, even if fungal growth was effectively inhibited**.**

This concentration-dependent behavior was mirrored in the material's physical stability. The CB5% group exhibited substantial mass loss (−4.18%), indicating hydrolytic degradation and plasticizer leaching from a poorly integrated composite structure (8). In contrast, the exceptional stability of the CB 1% and 2% formulations, with minimal mass change (∼ −0.6%), confirms that optimal loading enhances structural integrity by mitigating water uptake and promoting a homogeneous network.

Mechanically, CB incorporation significantly altered key properties. A plasticizing effect was observed at lower concentrations, reducing Shore A hardness. The shore A hardness of 51.3 in the CB1% incorporated silicone liners was close to the compliance standard of the ISO, as the upper limit is 50 (19). Interestingly, this trend reversed in the CB5% group, in which the tear strength peaked (5.31 ± 1.84 N/mm), surpassing the negative control group. This suggests a shift in mechanism, namely, at higher concentrations, boric acid likely facilitates ionic cross-linking within the PDMS network of the silicon network, thereby reinforcing the structure and enhancing resistance to tear propagation (24, 25). Conversely, tensile bond strength consistently decreased to below clinical thresholds (>0.44 MPa) with increasing CB concentration, falling to 0.19–0.24 MPa. This can be attributed to the surfactant nature of the ionic liquid, which disrupts adhesion at the silicone-acrylic interface, as consistently evidenced by the adhesive failure mode (26, 27). This indicates a fundamental incompatibility between the polydimethylsiloxane-based liner and the acrylic resin of the denture base, with separation occurring at the junction of the two materials (28).

The quantitative data from the Alamar Blue assay across all the experimental groups suggest that the CB-containing formulations supported cellular viability rather than inducing cytotoxic effects. The enhanced proliferation found in the CB1% and CB5% groups by day 7 indicates a potentially stimulatory effect at these concentrations, consistent with the dose-dependent biocompatibility reported for similar biomaterials in previous studies (29, 30). The absence of a comparable proliferation surge in the CB2% group may reflect a concentration-specific cellular response, where intermediate levels do not produce the same stimulatory effect as lower or higher concentrations.

Critically, all the CB-modified liners demonstrated favorable biocompatibility, significantly enhancing NIH3T3 fibroblast proliferation over a 7-day period without cytotoxicity.

This confirms that the chemical constituents, i.e., choline hydroxide and boric acid, are cytocompatible and that the modifications support cell growth, a prerequisite for any intraoral application (11, 12).

In conclusion, choline borate ionic liquid represents a promising strategy for developing bioactive silicone denture liners. The 1% and 2% concentrations offer the optimal balance, providing strong antifungal activity, superior structural stability, and biocompatibility, albeit with reduced hardness and bond strength.

## 5 Conclusion

The incorporation of choline borate into silicone denture liners at concentrations of 1% or 2% demonstrated a promising balance of properties, showing effective antifungal action and good biocompatibility alongside acceptable structural stability. However, this enhancement was accompanied by increased softness and a decrease in tensile bond strength below typical clinical standards. At a concentration of 5%, a decline in resistance to moisture-induced degradation, hardness, and tensile bond strength was observed. These initial, concentration-dependent findings imply that precise regulation of the choline borate concentration is essential to maximize its bioactive advantages without unduly jeopardizing the mechanical integrity of the liner. Given the *in vitro* nature of this study, the conclusions regarding clinical impact remain preliminary and warrant further validation through aging simulations and bond strength optimization.

### 5.1 Limitations of the study

The scope of this *in vitro* study has several limitations. The low tensile bond strength of all the tested formulations, below the empirical clinical threshold, indicates a need for mechanical optimization. While chemical incorporation was confirmed (FTIR/NMR), surface properties (roughness and wettability) and the long-term release kinetics of the ionic liquid after material aging remain uncharacterized. These concerns may be addressed in future work.

## Data Availability

The raw data supporting the conclusions of this article will be made available by the authors, without undue reservation.
