# Supplementary material for: Assessment of a novel antifungal ionic liquid-mediated silicone denture base soft liner
Source: Front Dent Med. 2026 Feb 18;7:1734528. doi: 10.3389/fdmed.2026.1734528 (PMC12957269; doi:10.3389/fdmed.2026.1734528)
Supplement: Supplementary file 1 [file Datasheet1.pdf]

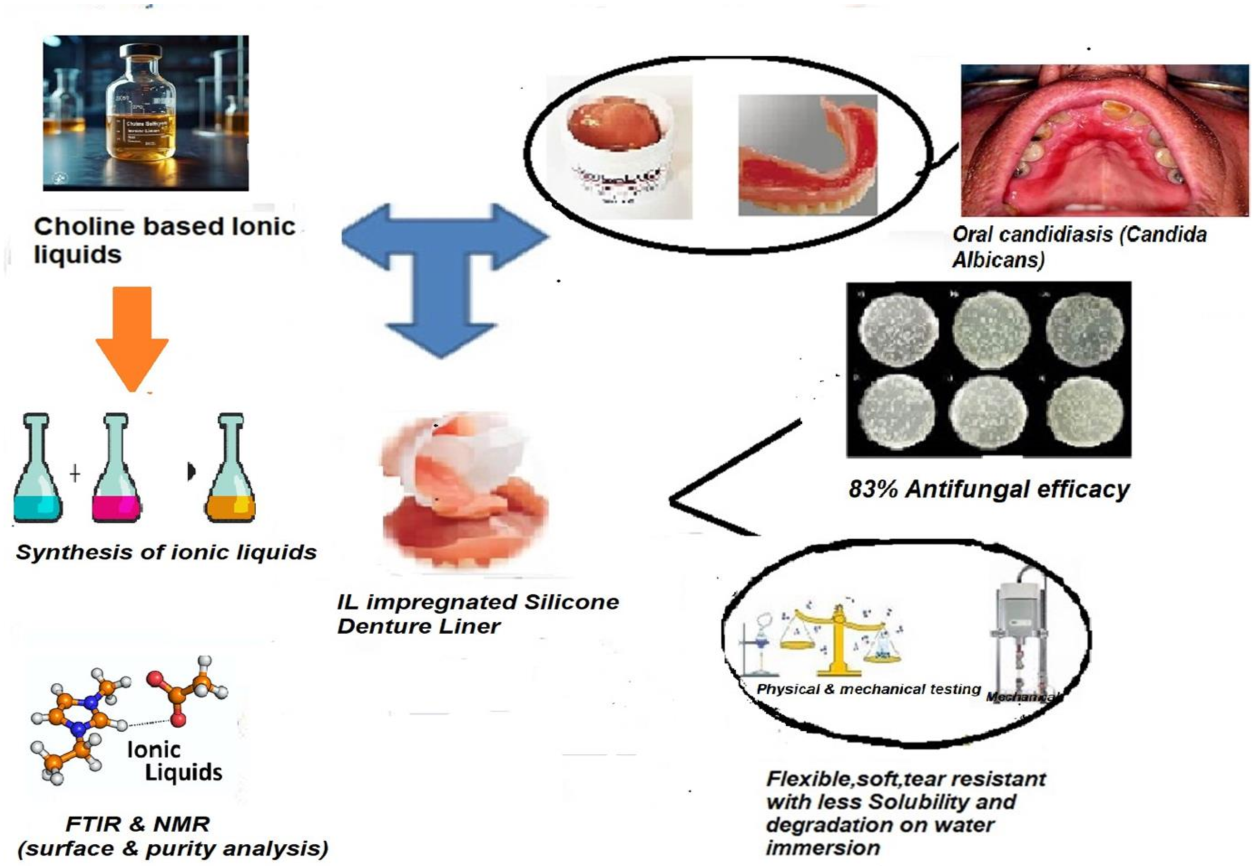

Choline based Ionic liquids
Synthesis of ionic liquids
Ionic Liquids
FTIR & NMR (surface & purity analysis)
IL impregnated Silicone Denture Liner
Oral candidiasis (Candida Albicans)
83% Antifungal efficacy
Physical & mechanical testing
Flexible,soft,tear resistant with less Solubility and degradation on water immersion
